# Alterations in gut microbiota and plasma metabolites: a multi-omics study of mild cognitive impairment in Parkinson’s disease

**DOI:** 10.3389/fnins.2025.1667331

**Published:** 2025-12-01

**Authors:** Zihao Lin, Yangdanyu Li, Yuning Liu, Bo Yang, Peixiao Yin, Chenyang Guan, Yating Fang, Liying Yang, Kun Zan, Guiyun Cui, Lu Yu, Xiaojie Wang, Chuanying Xu

**Affiliations:** 1 Department of Neurology, The Affiliated Hospital of Xuzhou Medical University, Xuzhou, Jiangsu, China; 2 Department of Neurology, The First Clinical College, Xuzhou Medical University, Xuzhou, Jiangsu, China; 3 Department of Pharmacy, The Affiliated Hospital of Xuzhou Medical University, Xuzhou, Jiangsu, China

**Keywords:** Parkinson’s disease with mild cognitive impairment, gut microbiota, plasma metabolites, 16S rRNA sequencing, LC-MS, gut-brain axis

## Abstract

**Introduction:**

Emerging evidence suggests that gut microbiota and plasma metabolites may be associated with the onset and progression of Parkinson’s disease (PD). The interplay between gut microbiota and plasma metabolites in influencing the progression of cognitive impairment in PD is yet to be fully understood and requires further exploration. Our objective was to investigate the roles of gut microbiota and plasma metabolites in PD cognitive impairment.

**Methods:**

We initially recruited 100 individuals with PD and 50 healthy controls (HCs). After excluding participants based on education level and cognitive screening criteria, the final cohort comprised 38 PD patients and 40 HCs. We examined fecal and plasma specimens from these participants. Cognitive function was assessed via the Montreal Cognitive Assessment (MoCA). Gut microbiota was analyzed through 16S rRNA sequencing, and plasma metabolites were evaluated via Liquid Chromatography–Mass Spectrometry (LC-MS). Using Spearman correlation to analyze the association between gut microbiota and plasma metabolites.

**Results:**

PD patients with mild cognitive impairment (PD-MCI) exhibited distinct microbial and metabolic profiles compared to PD patients with normal cognition (PD-NC). Consistent with both the Gut Microbiota Health Index (GMHI) and Gut Microbiota Health Index (MDI), PD-MCI patients exhibited significant gut microbial dysbiosis. Multi-algorithm differential abundance analysis identified *g__Eggerthella* as a core depleted genus in PD-MCI, consistently validated across both LEfSe and MaAsLin2 analyses. Additional microbial alterations included depletion of Short-Chain Fatty Acids (SCFA)-producing genera (*g__Blautia*, *g__Lachnoclostridium*, *g__Erysipelatoclostridium*, *g__norank_f__norank_o__Oscillospirales, g__Megasphaera,* and *g__Lactococcus*) and enrichment of *g__Senegalimassilia* in PD-MCI. Metabolite analysis revealed that phenylalanine metabolism (including phenylacetylglutamine, 2-hydroxycinnamic acid, N-acetyl-L-phenylalanine, and phenylacetylglycine) and PPAR signaling pathways (including 8-hydroxy-5Z,9E,11Z,14Z-eicosatetraenoic acid) were downregulated in the PD-MCI group, while choline metabolism in cancer (including PC(18:1(11Z)/18:3(6Z,9Z,12Z)) and LysoPC(18:3(6Z,9Z,12Z)/0:0)) was upregulated. Notably, phenylacetylglutamine demonstrated robust diagnostic potential (AUC = 0.8222), emerging as a promising biomarker for PD-MCI. Correlation analysis revealed significant associations between key microbial taxa (particularly *g__Eggerthella* and SCFA-producing genera) and metabolites (phenylacetylglutamine, and uridine 2′,3′-cyclic phosphate), suggesting their interactive role in PD cognitive impairment through gut-brain axis mechanisms.

**Conclusion:**

Our multi-omics study revealed distinct gut microbiota and metabolite alterations in PD patients with cognitive impairment, highlighting gut-brain axis dysfunction. Key microbial and metabolic markers demonstrated diagnostic potential, providing new insights into the pathophysiology of PD-related cognitive decline and potential targets for future therapeutic strategies.

## 1 Introduction

Parkinson’s disease (PD) represents a degenerative neurological disease marked by dopamine-producing nerve cell deterioration and Lewy protein aggregates accumulation (Yang et al., 2024a). The clinical manifestations of PD consist of two main types of symptoms: motor dysfunction, which is manifested by bradykinesia, muscle rigidity and resting tremor; and non-motor symptoms, which are mainly related to sleep disorders, autonomic dysfunction, mood abnormalities and cognitive decline (Beitz, 2014). Among these, cognitive impairment has attracted increasing attention due to its significant impact on patients’ quality of life. However, the mechanisms underlying its development remain poorly understood.

Current research suggests that the development of cognitive impairment in PD is associated with a number of factors, such as the build-up of beta-amyloid and tau proteins in the central nervous system, oxidative stress, neuroinflammation, traumatic brain injury, environmental factors (e.g., pesticide and tobacco exposure), and genetic risk factors (including genes such as COMT, APOE, MAPT, and BDNF) (Gonzalez-Latapi et al., 2021). Recent studies have demonstrated that the gut-brain axis exerts a substantial influence on the pathogenesis of PD. The metabolic processes of intestinal microorganisms play a vital role in regulating the host’s physiological functions and wellness (Mukherjee et al., 2016; Zhang et al., 2023). Similarly, changes in the gut microbial community have a significant impact on the development of Alzheimer’s disease (AD) (Jiang et al., 2017; Chandra et al., 2023). Given that PD patients often exhibit AD-related pathological features, such as beta-amyloid and tau protein deposits (Aarsland et al., 2021), it is plausible that these contribute to an accelerated cognitive decline. Consequently, the mechanism by which the gut-brain axis influences cognitive deficits in PD requires further exploration.

To better understand early mechanisms of cognitive decline while minimizing the impact of the complex dementia state and reducing confounding factors, this study focuses on PD patients with mild cognitive impairment (PD-MCI). We recruited 100 PD patients and 50 healthy controls (HCs) for fecal and plasma sample collection. Cognitive status was evaluated using Montreal Cognitive Assessment (MoCA) scale (Skorvanek et al., 2018). Subsequently, analysis of the gut microbiota was conducted via 16S rRNA gene sequencing, while plasma metabolite analysis was performed using liquid chromatography-mass spectrometry (LC-MS). Correlation analyses were conducted to identify potential associations between gut microbiota and metabolites.

## 2 Materials and methods

### 2.1 Participants

The study participants were recruited from the Affiliated Hospital of Xuzhou Medical University in Xuzhou, China, between May 2022 and June 2023. We initially recruited 100 PD patients and 50 HCs with no history of PD. All patients with PD met the International Parkinson and Movement Disorder Society clinical diagnostic criteria in 2015 (Postuma et al., 2015).

Participants were omitted from the study with: (a) diagnosed psychiatric disorders; (b) atypical or secondary parkinsonism; (c) antibiotic, probiotic, or prebiotic use within the last 1 month; (d) co-infection; (e) severe chronic acute illness or severe cognitive impairment that hindered their ability to complete the study questionnaires; (f) acute and chronic primary gastrointestinal disorders; and (g) DBS surgery. These exclusion criteria were formulated according to the patient’s clinical background and pertinent hospitalization records.

Following initial recruitment, participants underwent cognitive assessment. To minimize potential interference from insufficient education or illiteracy on the MoCA scale, we excluded 24 PD patients and 4 HCs with less than 6 years of education. Additionally, to focus on early-stage cognitive changes, we excluded participants with MoCA scores ≤ 20 points (38 PD patients and 6 HCs were excluded). After applying these criteria, the final study cohort consisted of 38 PD patients and 40 HCs. The PD patients were further classified into two groups: 18 with normal cognition (PD-NC; MoCA >25) and 20 with mild cognitive impairment (PD-MCI; MoCA 21–25).

Each patient provided informed consent. The study received ethical approval from the Ethics Committee of the Affiliated Hospital of Xuzhou Medical University (approval number: XYFY2022-KL262-01, approval date: 26 July 2022; approval number: XYFY2023-KL266-01, approval date: 02 August 2023). This investigation adhered to the Declaration of Helsinki principles.

### 2.2 Clinical data assessment

Clinical data evaluation was performed by ZH.L., YDY.L., YN.L. at the Affiliated Hospital of Xuzhou Medical University (Xuzhou, Jiangsu Province, China). Our study recorded demographic and clinical characteristics, comprising age, disease duration, gender, Body Mass Index (BMI), Levodopa Equivalent Daily Dose (LEDD), Hoehn and Yahr stage (H-Y stage), and years of education.

MoCA scale was utilized to assess the patients’ cognitive functions during the “on” period, patients scoring between 20 and 25 points were identified as PD-MCI, while those scoring 20 points or below were classified as Parkinson’s disease dementia (PDD) (Skorvanek et al., 2018). The Movement Disorder Society-Unified Parkinson’s Disease Rating Scale Part III (MDS-UPDRS III) was used to assess motor symptoms (Postuma et al., 2015).

### 2.3 Collection and processing of plasma and fecal specimens

Peripheral venous blood was collected from each participant. The samples were then centrifuged at 3000 × g for 10 min within an hour after collection. Fecal samples were obtained from subjects during the early hours of the day. Both plasma and fecal samples remained preserved at −80 °C pending analysis. Gut microbiota in fecal specimens were sequenced using 16S rRNA sequencing and metabolites contained in plasma specimens were analyzed using LC-MS.

### 2.4 Statistical analysis

Continuous demographic variables appear as mean (standard deviation) or median (interquartile range), while categorical variables are shown as percentages. For two-group comparisons, Student’s *t*-test was used for normal distributions and Mann–Whitney U-test for non-normal distributions. For three-group comparisons, one-way ANOVA was used for normal distributions and Kruskal-Wallis test for non-normal distributions. Chi-square test was used for categorical data comparisons. Statistical significance was defined as *p* < 0.05, with analyses conducted using SPSS (version 29.0).

Calculating the alpha-diversity indices with Mothur v1.30.2 (Schloss et al., 2009), the Kruskal-Wallis rank sum test was used to compare the differences between groups. Microbial community similarities between samples were assessed via Principal Coordinate Analysis (PCoA) using both unweighted and weighted UniFrac in the Vegan v2.4.3 software package. And to compare between-group differences using Adonis. Beta-diversity indices differences between groups were analyzed using Partial Least Squares Discriminant Analysis (PLS-DA). To evaluate within-group dispersion, we calculated each sample’s Bray–Curtis dissimilarity distance to its group centroid (geometric median) and compared these distance-to-centroid values between groups using the Wilcoxon rank-sum test in R (version 3.3.1) (Abbas et al., 2019). The Gut Microbiota Health Index (GMHI) and the Microbial Dysbiosis Index (MDI) were applied to further evaluate the gap in gut microbiota between the cohorts. It distinguishes between healthy and unhealthy populations more effectively than alpha-diversity indices and ecological measures like Shannon diversity and richness, with consistent stratification performance across populations and it is generally regarded as an indicator of gastrointestinal health and the presence of dysbiosis (Gupta et al., 2020). MDI is an index used to assess the degree of microbial ecological disturbance, with higher scores indicating more severe disturbance (Gunathilake et al., 2020). The Linear Discriminant Analysis (LDA) effect size (LEfSe) (Segata et al., 2011), Microbiome Multivariable Associations with Linear Models 2 (MaAsLin2) (Mallick et al., 2021), and Microbiome Multivariable Associations with Linear Models 3 (MaAsLin3) (Nickols et al., 2024) were utilized to identify significantly enriched bacterial taxa (from phylum to genus) in different groups. LEfSe analysis was performed with LDA scores > 2 and *p* < 0.05 as significance thresholds. MaAsLin2 and MaAsLin3 analyses were conducted with adjusted *p* < 0.05 after adjusting for potential confounders including age, gender, BMI, LEDD, UPDRS III score, year of education and disease duration. Microbial taxa identified by at least two algorithms were considered as core differential microbes.

**Figure 1:**
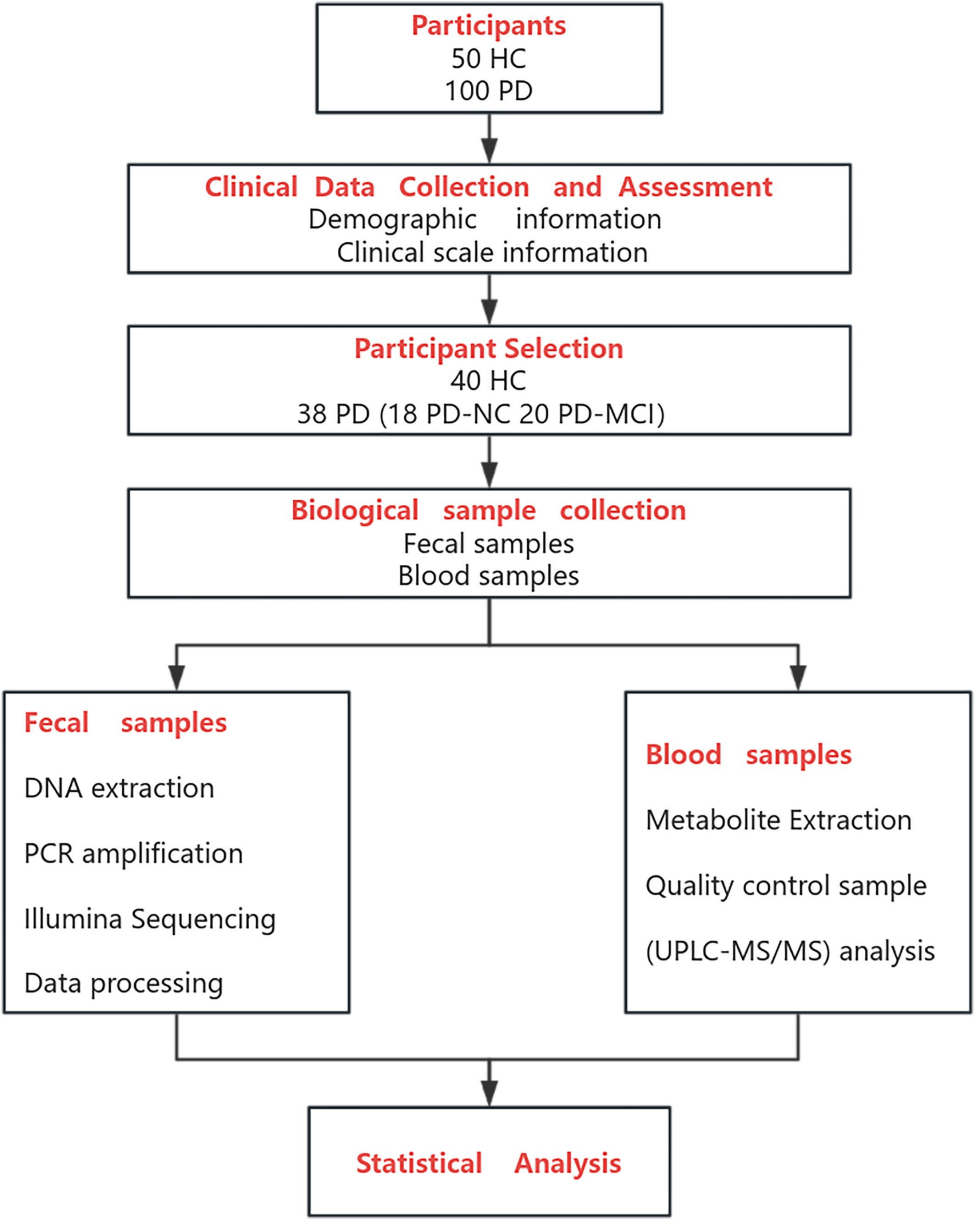
The flow chart of the section of materials and methods.

The R package ropls (version 1.6.2) was used for Principal Component Analysis (PCA) and PLS-DA. Notable distinct metabolic compounds were identified through Variable Importance in Projection (VIP) values (VIP > 1) derived from PLS-DA analysis and confirmed via Student’s *t*-test with statistical threshold of *p* < 0.05. Using the Kyoto Encyclopedia of Genomes (KEGG) database, distinguishing metabolic compounds between the comparison groups were linked to their biological pathway through pathway enrichment evaluation. Metabolite enrichment analysis conducted via the “scipy.stats” package in Python. Assessment of Metabolite Diagnostic Performance via Receiver Operating Characteristic (ROC) Analysis. Correlations between differentially expressed metabolites and differentially abundant gut microbiota (genus level) were evaluated using Spearman correlation coefficients, with statistical significance set at *p* < 0.05 (Figure 1).

## 3 Results

### 3.1 Demographic and clinical characteristics of participants

Our study initially enrolled 100 individuals with PD alongside 50 healthy participants of comparable age. Subsequent to the implementation of exclusion criteria (education <6 years and MoCA scores ≤20), the final cohort comprised 38 PD patients (18 PD-NC and 20 PD-MCI) and 40 HCs. No statistically significant differences were observed among groups (PD vs. HC, and PD-MCI vs. PD-NC) in terms of age, gender, years of education, disease duration, LEDD, MDS-UPDRS III, H-Y stage and BMI (Table 1).

**Table 1:** Demographic and clinical characteristics of participants.

Characteristics	PD-NC	PD-MCI	HC	P-adjust
Age (years)	63.0 (53.0, 69.8)	60.0 (57.0, 64.8)	58.0 (55.0, 67.2)	0.718
Male sex, *n* (%)	10 (55.6%)	12 (60.0%)	17 (42.5%)	0.382
Education, years	12 (9, 12)	9 (9, 15)	12 (9, 12)	0.683
BMI, Kg/m^2^	22.9 ± 3.6	24.6 ± 3.5	24.8 ± 3.2	0.136
MoCA score	26 (26, 28)	23 (22, 24)	25 (22, 26)	<0.001
Disease duration, years	4 (2, 5)	5 (3, 7)	NA	0.411
LEDD (mg)	635.8 ± 357.7	464.5 ± 332.8	NA	0.135
MDS-UPDRS III score	26 (22, 43)	30 (22, 39)	NA	1.000
H-Y stage	2.5 (2, 2.5)	2 (2, 2.5)	NA	0.381

LEDD, Levodopa Equivalent Daily Dose; MDS-UPDRS III, Movement Disorder Society Unified Parkinson’s Disease Rating Scale Part III; MoCA, Montreal Cognitive Assessment; PD-NC, Parkinson’s disease with no cognitive impairment; PD-MCI, Parkinson’s disease with mild cognitive impairment; HC, healthy control group.

### 3.2 Fecal microbiota analysis

To characterize the microbial community richness and diversity among HC, PD-NC and PD-MCI groups, we performed comprehensive alpha-diversity indices analyses utilizing multiple ecological indices, including Sobs, Shannon, Chao, ACE, Simpson, and coverage. Statistical analyses revealed no significant differences in any of the aforementioned alpha-diversity indices between the PD-MCI and PD-NC groups (Table 2). These results indicate that there were no differences in gut microbiota diversity or richness among HC, PD-NC and PD-MCI groups.

**Table 2:** Alpha diversity indices analyses.

Estimators	HC-Mean	HC-Sd	PD-MCI-Mean	PD-MCI-Sd	PD-NC-Mean	PD-NC-Sd	*p*-value	P-adjust
Sobs	240.2	87.493	269.35	76.736	250.28	93.97	0.5224	0.5224
Shannon	2.9094	0.71008	3.2713	0.66027	3.1761	0.69368	0.1394	0.423
Simpson	0.15243	0.11938	0.11438	0.10156	0.11136	0.08469	0.1952	0.423
Ace	290.76	103.3	335.15	94.522	300.96	113.62	0.269	0.423
Chao	291.16	102.92	333.3	93.854	300.46	118.21	0.2957	0.423
Coverage	0.99745	0.00096	0.99711	0.00095	0.9975	0.00116	0.3525	0.423

PD-NC, Parkinson’s disease with no cognitive impairment; PD-MCI, Parkinson’s disease with mild cognitive impairment; HC, healthy control group.

**Figure 2:**
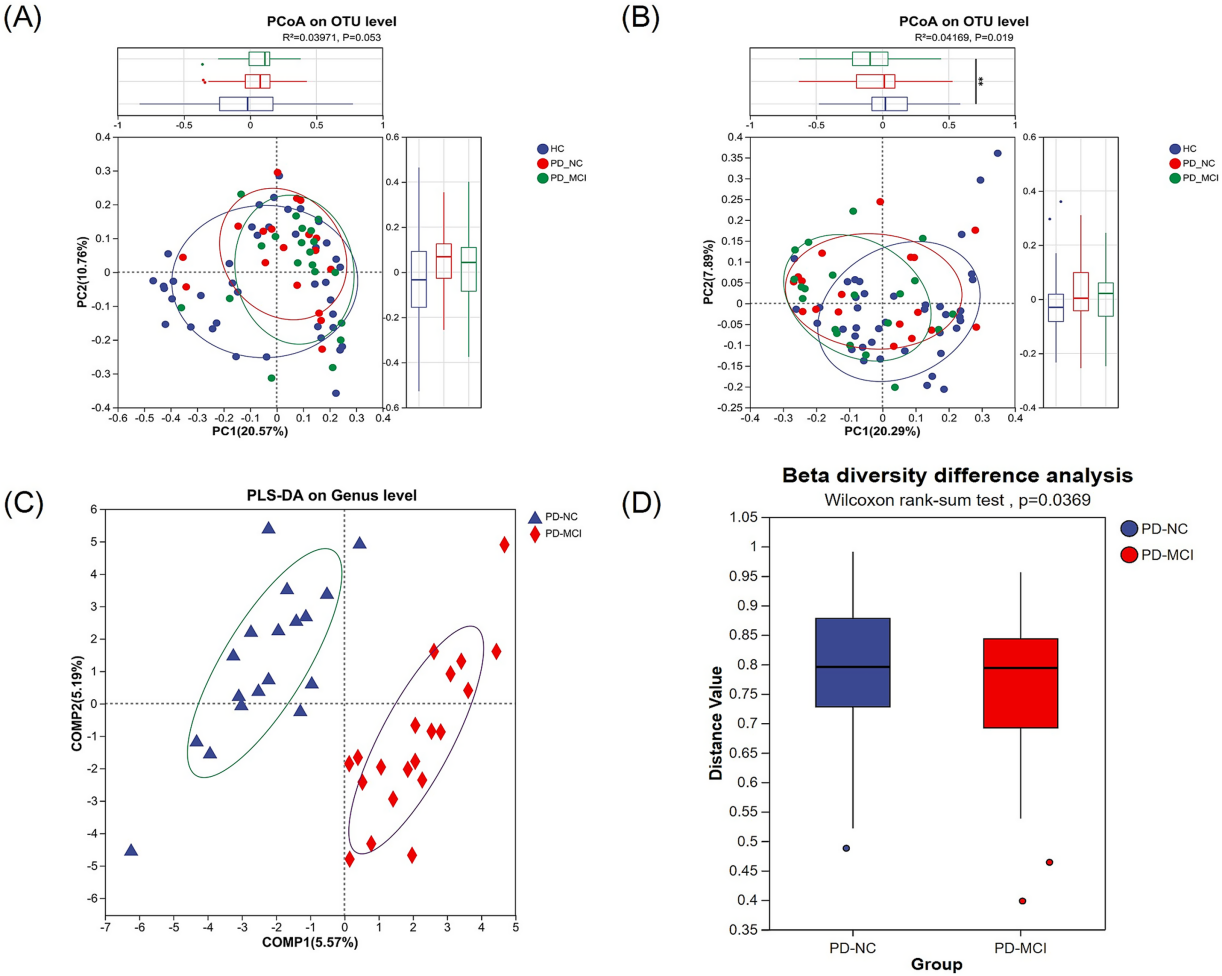
**(A,B)** PCoA based on the weighted normalized UniFrac distance **(A)** and the unweighted UniFrac distance **(B)**. Each point represents one sample, and samples from the same group are clustered together. The percentage in parentheses along the axes indicates the explained variance in species composition. **(C)** PLS-DA separates the two groups (PD-MCI vs. PD-NC) based on beta-diversity indices. **(D)** Wilcoxon rank sum test for differences in beta-diversity indices shows significant differences between the PD-MCI and PD-NC groups (adjusted *p* = 0.037).

In parallel, to examine the microbial community structure among HC, PD-NC and PD-MCI groups, we performed beta-diversity indices analysis using PCoA. While weighted UniFrac-based PCoA showed no clear separation among groups (*R*^2^ = 0.03971, *p* = 0.053, Figure 2A), unweighted UniFrac-based PCoA revealed significant compositional differences (*R*^2^ = 0.04169, *p* = 0.019, Figure 2B), particularly along PC1 axis (*p* = 0.005). To further discriminate between PD-NC and PD-MCI groups, we performed PLS-DA at the genus level. The PLS-DA successfully distinguished the two groups, demonstrating clear separation between PD patients with and without cognitive impairment (Figure 2C). The PLS-DA loading plot (Supplementary Figure S1) reveals the key gut microbial taxa contributing to the separation between PD-MCI and PD-NC groups, with taxa exhibiting high loading values representing the most discriminative features. Furthermore, to assess within-group heterogeneity, we calculated each sample’s Bray–Curtis dissimilarity to its respective group centroid. Wilcoxon rank-sum test comparing these distance-to-centroid values revealed significant differences between groups (adjusted *p* = 0.037, Figure 2D), these results suggest differences in both gut microbiota structure and internal variability between PD-MCI and PD-NC groups.

**Figure 3:**
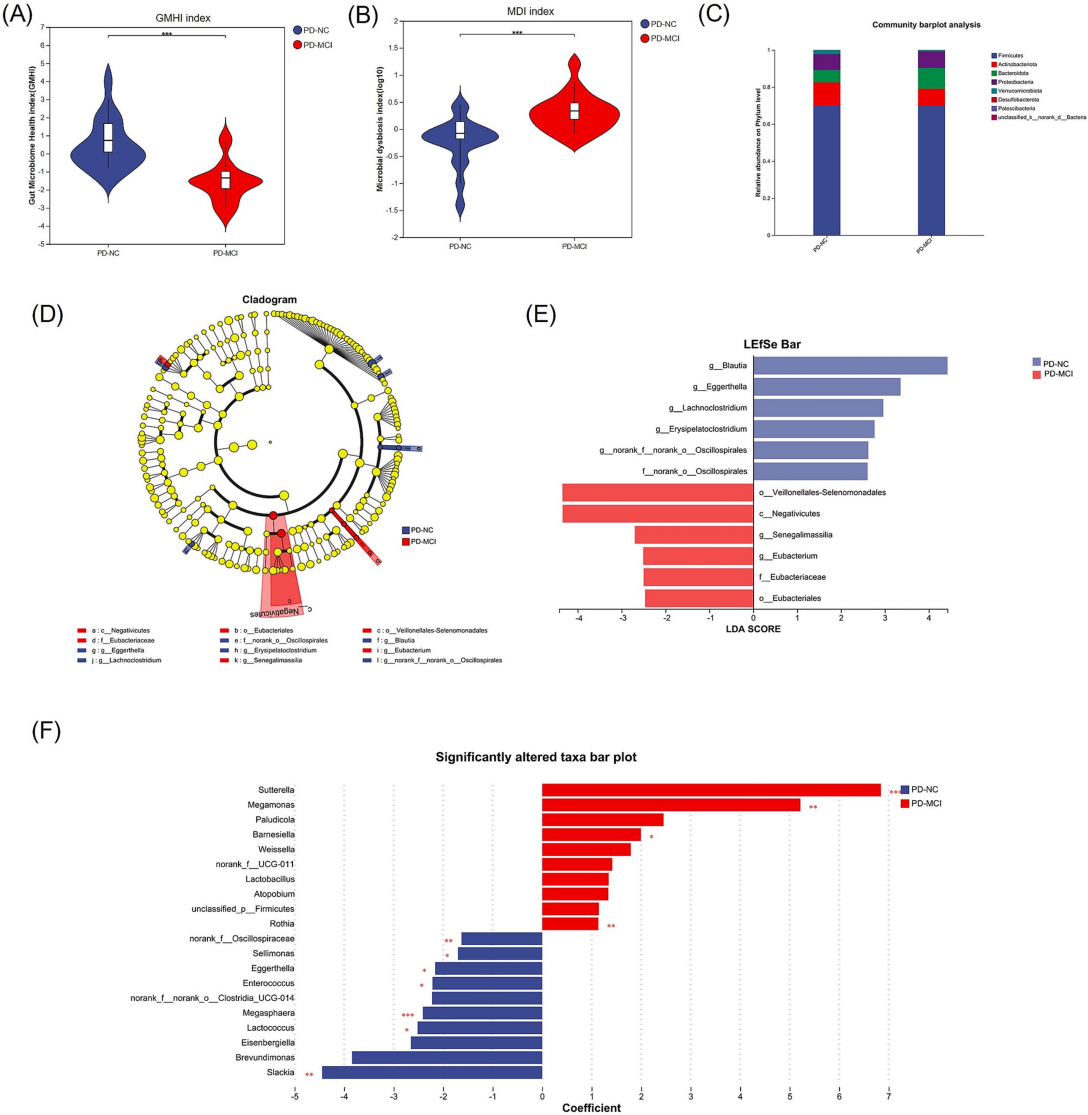
**(A)** Comparison of the MDI between the PD-MCI and PD-NC groups, showing a significant difference in microbial imbalance. **(B)** Comparison of the GMHI between the PD-MCI and PD-NC groups, indicating a marked disparity in overall gut health status. **(C)** Community bar plot analysis at the phylum level reveals compositional differences in gut microbiota between the two groups. **(D)** LEfSe-based cladogram illustrating the significantly different taxa between PD-MCI and PD-NC. Nodes highlighted in color represent taxa enriched in each group. **(E)** LDA bar chart generated by LEfSe for the two groups. **(F)** MaAsLin2 analysis showing regression coefficients for significantly altered taxa. * Adjusted *p* < 0.05, ** adjusted *p* < 0.01, *** adjusted *p* < 0.001.

Given these structural differences, we further analyzed both GMHI and MDI to comprehensively evaluate the gut microbiota health status, among HC, PD-NC and PD-MCI groups. GMHI analysis revealed significantly higher scores in the PD-NC group compared to the PD-MCI group (adjusted *p* < 0.001, Figure 3A). Similarly, in the comparison between HC and both PD-NC and PD-MCI groups, HC exhibited significantly higher scores (adjusted *p* < 0.001, Supplementary Figures S2A,B). Further analysis revealed significantly elevated MDI values in the PD-MCI group compared to the PD-NC group (adjusted *p* < 0.001, Figure 3B). We also analyzed the MDI differences between both PD-NC and PD-MCI groups versus HC. We found that both PD-NC and PD-MCI groups exhibited higher MDI values compared to the HC group (adjusted *p* < 0.001, Supplementary Figures S2C,D). Collectively, these findings suggest that PD-NC group maintain a more favorable gut microbiota profile compared to PD-MCI group.

To better understand the compositional differences, we investigated the taxonomic composition of the gut microbiota between PD-MCI and PD-NC groups. At the phylum level, comparative analysis showed significant differences in microbial abundances between groups. Specifically, *p__Bacteroidota* and *p__Proteobacteria* showed higher relative abundances in the PD-MCI group, whereas *p__Firmicutes*, *p__Actinobacteriota*, and *p__Verrucomicrobiota* were reduced compared to the PD-NC group (Figure 3C). To further elucidate these taxonomic differences at a finer resolution, we performed LEfSe, MaAslin2, and MaAslin3 analyses at the genus level. LEfSe analysis identified differential microbiota, with *g__Blautia*, *g__Eggerthella*, *g__Lachnoclostridium*, *g__Erysipelatoclostridium*, and *g__norank_f__norank_o__Oscillospirales* being significantly enriched in the PD-NC, while *g__Senegalimassilia* and *g__Eubacterium* were significantly more abundant in the PD-MCI (Figures 3D,E). MaAsLin2 identified 11 differentially abundant genera between PD-MCI and PD-NC groups (FDR < 0.05, Figure 3F). Among these, 4 genera were significantly enriched in PD-MCI: *g__Sutterella*, *g__Megamonas*, *g__Barnesiella*, and *g__Rothia*. Conversely, 7 genera showed significant enrichment in PD-NC group: *g__Sellimonas*, *g__norank_f__Oscillospiraceae, g__Eggerthella, g__Enterococcus, g__Megasphaera, g__Lactococcus,* and *g__Slackia.* We additionally performed MaAsLin3 analysis to further validate our findings. Under the stringent criterion of FDR < 0.05, no differentially abundant genera were identified. However, when applying a less conservative threshold of *p* < 0.05, *g__Senegalimassilia* emerged as significantly enriched in the PD-MCI group (Supplementary Table S1). Among these, *g__Eggerthella* was identified as a differential microorganism by both MaAsLin2 and LEfSe, and was therefore considered a key taxon.

### 3.3 Plasma metabolites analysis

**Figure 4:**
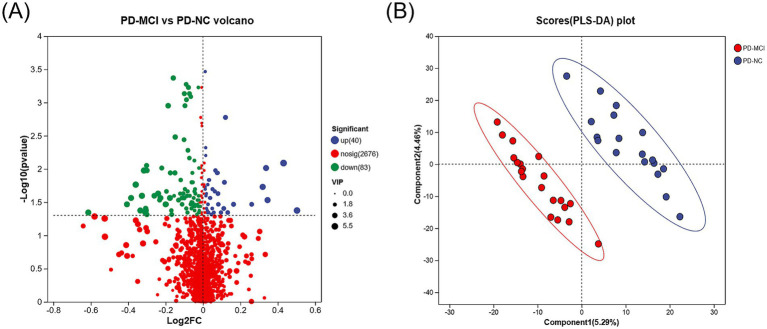
**(A)** Volcano plot illustrating the differentially expressed metabolites between the PD-MCI and PD-NC groups. The x-axis represents the log2 fold change, and the y-axis indicates the −log₁₀ (*p*-value). Blue dots denote significantly up-regulated metabolites, green dots represent significantly down-regulated metabolites, and red dots signify metabolites with no significant change. **(B)** PLS-DA score plot comparing the PD-MCI and PD-NC groups. Each point represents an individual sample.

To identify plasma metabolic changes and potential diagnostic markers in PD-MCI, we performed untargeted metabolites analysis on plasma samples from PD-MCI group and PD-NC group. Using PLS-DA and VIP analysis, we identified 123 differential metabolites between PD-MCI group and PD-NC group (VIP >1, *p* < 0.05). Among these metabolites, 40 were increased and 83 were decreased in the PD-MCI group compared to the PD-NC group (Figure 4). These metabolic alterations suggest significant metabolic disturbances in PD-MCI patients.

**Figure 5:**
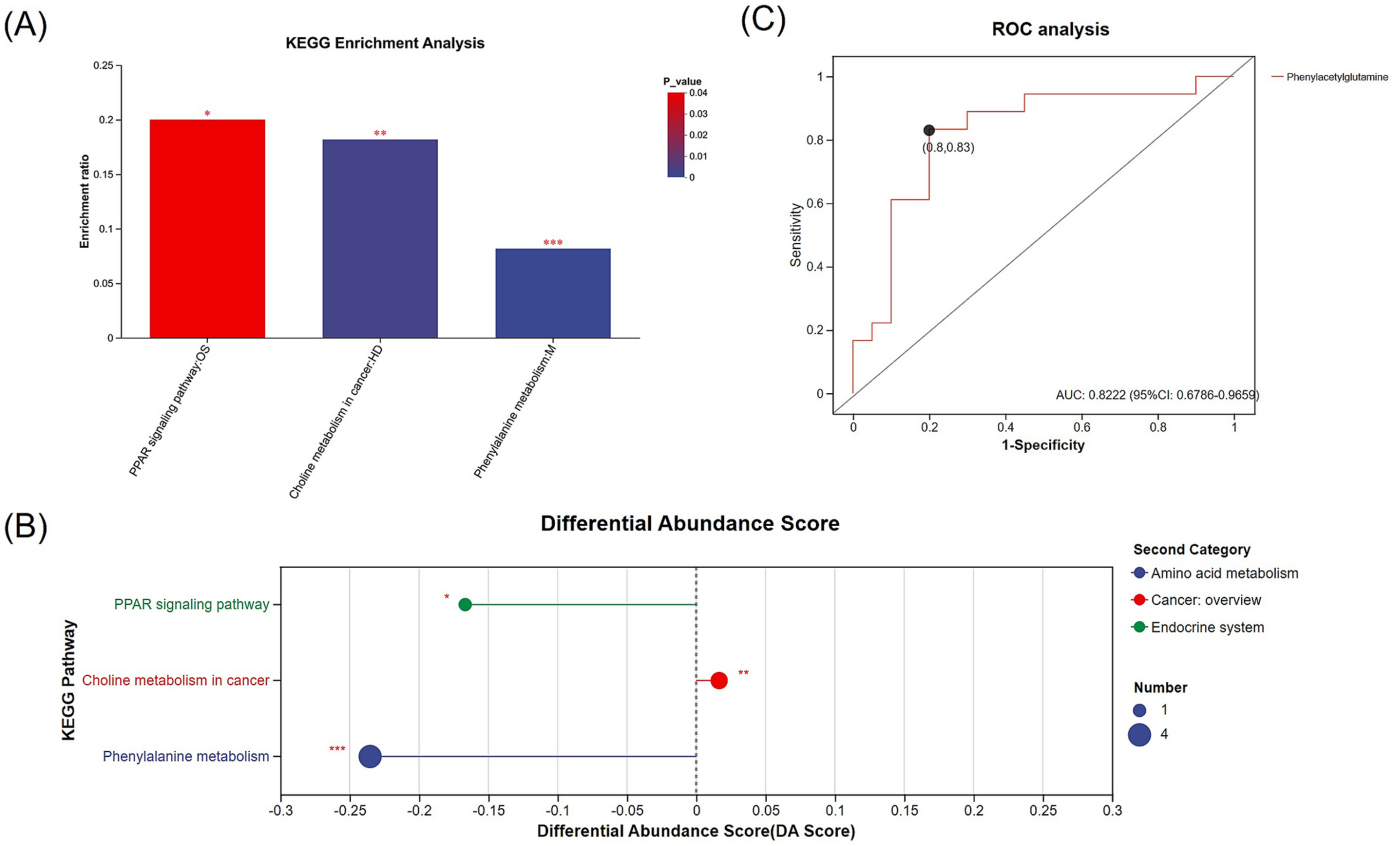
**(A)** KEGG enrichment analysis of differentially expressed metabolites between the PD-MCI and PD-NC groups, showing significantly enriched metabolic pathways. The x-axis represents the pathway name, and the y-axis indicates the enrichment ratio. Bars are color-coded according to the *p*-value. **(B)** Bubble plot illustrating the KEGG-enriched pathways, in which bubble size corresponds to the number of metabolites, and bubble color represents the second-level KEGG categories (e.g., amino acid metabolism, cancer overview, endocrine system). The x-axis indicates the differential abundance score (DA score), while the y-axis lists the significantly enriched pathways. **(C)** ROC analysis of the differentially expressed metabolite (phenylacetylglutamine) identified through KEGG enrichment (AUC = 0.8222). * *p* < 0.05, ** *p* < 0.01, *** *p* < 0.001.

Further analysis revealed 30 metabolites with biological significance in the KEGG database. KEGG enrichment analysis showed that these metabolites were significantly enriched in 3 metabolic pathways: phenylalanine metabolism (*p* < 0.001), choline metabolism in cancer (*p* = 0.002), and PPAR signaling pathway (*p* = 0.034) (Figures 5A,B). Among these, the metabolites in the phenylalanine metabolism and PPAR signaling pathway were downregulated in the PD-MCI group, while choline metabolism in cancer was upregulated. KEGG pathway enrichment discovered 7 key metabolites: 8-hydroxy-5Z,9E,11Z,14Z-eicosatetraenoic acid, 2-hydroxycinnamic acid, N-acetyl-L-phenylalanine, phenylacetylglycine, phenylacetylglutamine, PC(18:1(11Z)/18:3(6Z,9Z,12Z)), and LysoPC(18:3(6Z,9Z,12Z)/0:0) (Table 3). Among them, the first 5 metabolites significantly decreased in the PD-MCI group compared to the PD-NC group, while the latter 2 metabolites increased. Among these metabolites, 8-hydroxy-5Z,9E,11Z,14Z-eicosatetraenoic acid belongs to the PPAR signaling pathway; PC(18:1(11Z)/18:3(6Z,9Z,12Z)) and LysoPC(18:3(6Z,9Z,12Z)/0:0) are involved in choline metabolism in cancer; 2-hydroxycinnamic acid, N-acetyl-L-phenylalanine, phenylacetylglycine, and phenylacetylglutamine are associated with phenylalanine metabolism.

**Table 3:** KEGG pathway enrichment discovered 7 key metabolites.

Metabolite	Regulate (PD-MCI vs. PD-NC)	KEGG pathway description
8-Hydroxy-5Z,9E,11Z,14Z-eicosatetraenoic acid	Down	PPAR signaling pathway
2-Hydroxycinnamic acid	Down	Phenylalanine metabolism
N-Acetyl-L-phenylalanine	Down	Phenylalanine metabolism
Phenylacetylglycine	Down	Phenylalanine metabolism
PC(18:1(11Z)/18:3(6Z,9Z,12Z))	Up	Choline metabolism in cancer
LysoPC(18:3(6Z,9Z,12Z)/0:0)	Up	Choline metabolism in cancer
Phenylacetylglutamine	Down	Phenylalanine metabolism

KEGG, Kyoto Encyclopedia of Genes and Genomes.

ROC analysis was conducted on 7 distinct metabolites. The analysis revealed varying degrees of diagnostic potential: 8-hydroxy-5Z,9E,11Z,14Z-eicosatetraenoic acid (AUC = 0.7139), 2-hydroxycinnamic acid (AUC = 0.7000), N-acetyl-L-phenylalanine (AUC = 0.6917), phenylacetylglycine (AUC = 0.6889), PC(18:1(11Z)/18:3(6Z,9Z,12Z)) (AUC = 0.7222), and LysoPC(18:3(6Z,9Z,12Z)/0:0) (AUC = 0.7333) (Table 4). Notably, phenylacetylglutamine emerged as the most promising single biomarker for PD-MCI identification, demonstrating superior diagnostic capability (AUC = 0.8222) (Table 4; Figure 5C).

**Table 4:** ROC analysis results statistical table.

Metabolite	AUC	CI
2-Hydroxycinnamic acid	0.7000	[0.5254, 0.8746]
8-Hydroxy-5Z,9E,11Z,14Z-eicosatetraenoic acid	0.7139	[0.5442, 0.8836]
PC(18:1(11Z)/18:3(6Z,9Z,12Z))	0.7222	[0.5556, 0.8888]
Phenylacetylglycine	0.6889	[0.5123, 0.8655]
Phenylacetylglutamine	0.8222	[0.6786, 0.9659]
LysoPC(18:3(6Z,9Z,12Z)/0:0)	0.7333	[0.5625, 0.9041]
N-Acetyl-L-phenylalanine	0.6917	[0.5177, 0.8656]

AUC, the area under the curve; CI, confidence interval.

### 3.4 Correlation analysis of gut microbiota and plasma metabolites between the PD-MCI and PD-NC groups

**Figure 6:**
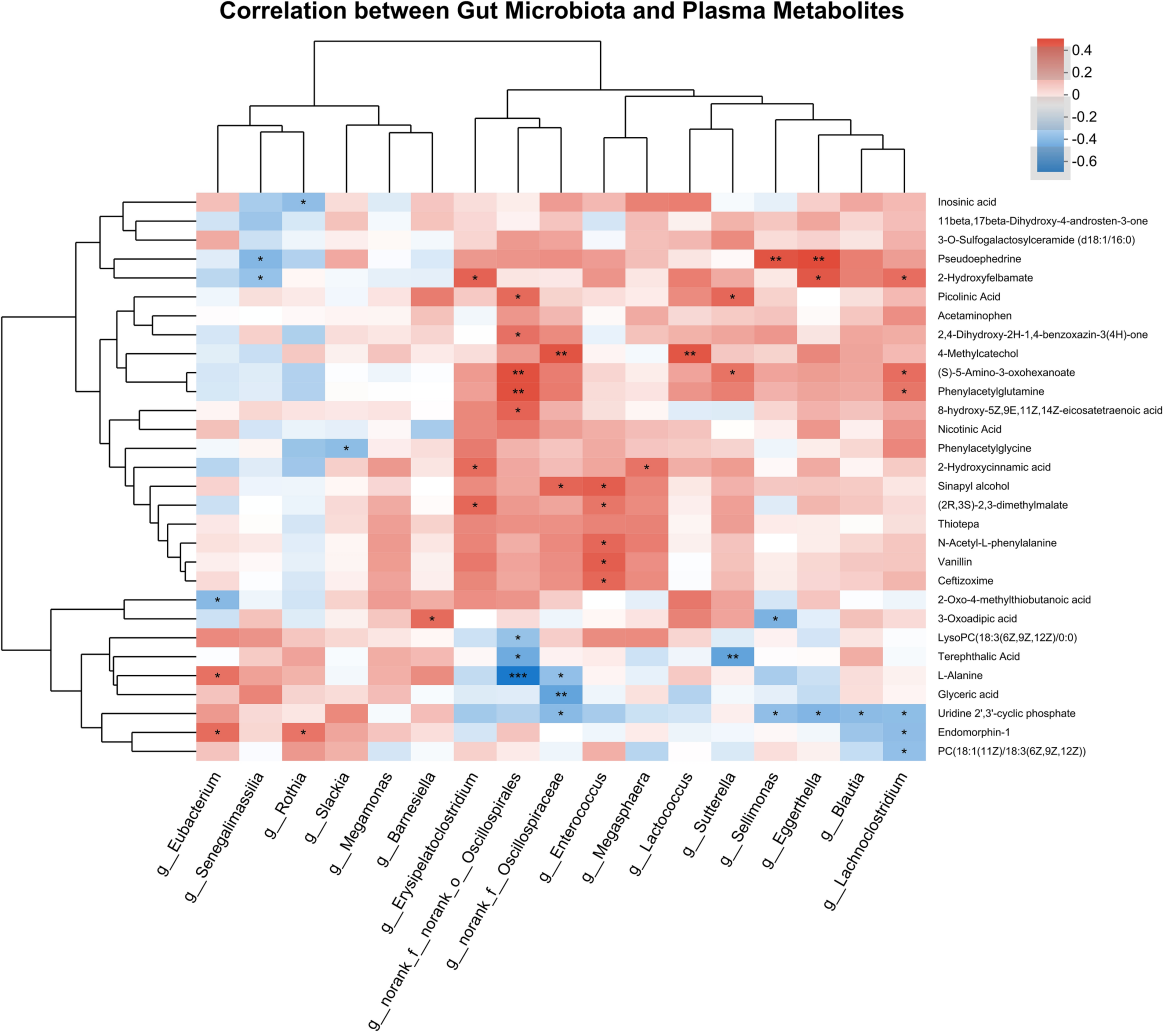
Heatmap showing the correlation between gut microbiota (genus level, selected by LEfSe and MaAsLin2) and the differentially expressed metabolites identified through KEGG pathway analysis in PD-MCI vs. PD-NC. Each row represents a metabolite, and each column denotes a bacterial taxon. Red cells indicate positive correlations, blue cells indicate negative correlations, and the intensity of the color corresponds to the magnitude of the correlation coefficient. Hierarchical clustering on both axes demonstrates the similarity among metabolites and bacterial taxa. * *p* < 0.05, ** *p* < 0.01, *** *p* < 0.001.

Spearman correlation analysis was conducted between KEGG-annotated differential metabolites and differentially abundant bacterial genera (genus level) identified by LEfSe (LDA > 2.0, *p* < 0.05) and MaAsLin2 (adjusted *p* < 0.05) between PD-NC and PD-MCI groups. This approach aimed to elucidate specific associations between bacterial genera and metabolites related to cognitive decline in PD, as visualized in Figure 6. In the PD-MCI group, *g__Senegalimassilia* negatively correlated with 2-hydroxyfelbamate and pseudoephedrine, *g__Eubacterium* showed a negative correlation with 2-oxo-4-methylthiobutanoic acid and positive correlations with L-Alanine and endomorphin-1. *g__Sutterella* positively correlated with picolinic acid and (S)-5-Amino-3-oxohexanoate but negatively correlated with terephthalic acid. *g__Barnesiella* exhibited a positive correlation with 3-oxoadipic acid, and *g__Rothia* negatively correlated with inosinic acid while positively correlating with endomorphin-1. In the PD-NC group, *g__Eggerthella* exhibited opposing correlations with uridine 2′,3′-cyclic phosphate, while positive correlations with pseudoephedrine and 2-hydroxyfelbamate. *g__Blautia* negatively correlated with uridine 2′,3′-cyclic phosphate. *g__Lachnoclostridium* positively correlated with 2-hydroxyfelbamate, (S)-5-amino-3-oxohexanoate, and phenylacetylglutamine, while showing negative correlations with uridine 2′,3′-cyclic phosphate, endomorphin-1, and PC(18:1(11Z)/18:3(6Z,9Z,12Z)). Additionally, *g__Erysipelatoclostridium* positively correlated with 2-hydroxyfelbamate, 2-hydroxycinnamic acid, and (2R,3S)-2,3-dimethylmalate. *g__norank_f__norank_o__Oscillospirales* showed positive correlations with multiple metabolites including 8-hydroxy-5Z,9E,11Z,14Z-eicosatetraenoic acid, (S)-5-amino-3-oxohexanoate, picolinic acid, 2,4-Dihydroxy-2H-1,4-benzoxazin-3(4H)-one and phenylacetylglutamine, while negatively correlating with L-Alanine, LysoPC(18:3(6Z,9Z,12Z)/0:0) and terephthalic acid. *g__Sellimonas* negatively correlated with uridine 2′,3′-cyclic phosphate and 3-oxoadipic acid, but positively correlated with pseudoephedrine. *g__norank_f__Oscillospiraceae* negatively correlated with L-alanine, glyceric acid, and uridine 2′,3′-cyclic phosphate, while positively correlating with sinapyl alcohol and 4-methylcatechol. *g__Enterococcus* positively correlating with (2R,3S)-2,3-dimethylmalate, sinapyl alcohol, N-acetyl-L-phenylalanine, vanillin, and ceftizoxime. *g__Megasphaera* positively correlated with 2-hydroxycinnamic acid, and *g__Slackia* negatively correlated with phenylacetylglycine.

## 4 Discussion

Through this multi-dimensional omics investigation, we characterized gut microbiota configurations and plasma metabolite profiles associated with PD-MCI. Our multi-algorithm approach (LEfSe, MaAsLin2, and MaAsLin3) combined with metabolomics analysis revealed several key observations: First, PD-MCI patients showed gut dysbiosis characterized by altered microbial diversity indices (GMHI and MDI) and specific taxonomic shifts. Second, the metabolite profile demonstrated expression changes in phenylalanine metabolism, choline metabolism, and PPAR signaling pathway, with phenylacetylglutamine showing potential as a biomarker (AUC = 0.8222). Third, correlation analyses indicated associations between specific bacterial genera and plasma metabolites, suggesting their potential involvement in PD-related cognitive impairment. These findings contribute to our understanding of the gut-brain axis in PD-MCI pathogenesis.

We evaluated both the alpha- and beta-diversity indices between the comparison cohorts, The PCoA analysis using weighted UniFrac distance showed no significant separation among groups, whereas unweighted UniFrac-based PCoA revealed significant compositional differences, particularly along the PC1 axis, indicating distinct microbial community structures between HC, PD-MCI and PD-NC groups. These findings suggest that while the overall abundance-weighted community structure appears similar, the presence/absence patterns of bacterial taxa differ significantly between groups, reflecting compositional shifts in rare or low-abundance species. The PLS-DA analysis revealed a clear distinction between the PD-MCI and PD-NC groups. Furthermore, our analyses of the GMHI and MDI indices demonstrated significant differences, underscoring the distinct microbial community profiles between the groups. The GMHI and MDI indices effectively serve as robust indicators of gut microbial health and the extent of dysbiosis. The significant decrease in GMHI in the PD-MCI group suggests that patients with more pronounced cognitive impairment have reduced gut microbiota health and greater microbiota imbalances associated with pathological states (Gupta et al., 2020). Meanwhile, the higher MDI in PD-MCI group may be a consequence of increased diversity of unhealthy-associated microbiota and ecological imbalance (Gunathilake et al., 2020).

To further investigate differential microbiota between the two groups, we employed multiple algorithms for differential microbial screening, including LEfSe, MaAsLin2, and MaAsLin3. LEfSe analysis revealed that at the genus level, *g__Blautia*, *g__Eggerthella*, *g__Lachnoclostridium*, *g__Erysipelatoclostridium*, and *g__norank_f__norank_o__Oscillospirales* were enriched in the PD-NC group, *g__Senegalimassilia* and *g__Eubacterium* were enriched in the PD-MCI group. MaAsLin2 analysis identified *g__Sellimonas, g__norank_f__Oscillospiraceae, g__Eggerthella, g__Enterococcus, g__Megasphaera, g__Lactococcus,* and *g__Slackia* as enriched in the PD-NC group, *g__Sutterella, g__Megamonas, g__Barnesiella,* and *g__Rothia* were enriched in the PD-MCI group. Notably, both algorithms converged on identifying *g__Eggerthella* as a common differential microorganism significantly enriched in the PD-NC group. Accumulating evidence has shown that *g__Eggerthella* is involved in the peripheral metabolism of levodopa prior to its passage across the blood–brain barrier, consequently compromising therapeutic outcomes in patients with PD (Huang et al., 2023; Xu et al., 2023). However, the potential role of *g__Eggerthella* in modulating cognitive function in remains to be elucidated in subsequent investigations. Although LEfSe and MaAsLin2 identified different differential microbiota, there exists an underlying functional connection. Notably, genera enriched in the PD-NC group, including *g__Blautia*, *g__Lachnoclostridium*, *g__Erysipelatoclostridium*, and *g__norank_f__norank_o__Oscillospirales* (identified by LEfSe), as well as *g__Megasphaera, g__Lactococcus* (identified by MaAsLin2), are collectively recognized as short-chain fatty acid (SCFA)-producing microorganisms (Hashizume et al., 2003; Konikoff and Gophna, 2016; Feng et al., 2020; Liu et al., 2021; Jiang et al., 2023; Cao et al., 2024; Otomo et al., 2025). These SCFA-producing microorganisms have demonstrated significant implications in the pathogenesis of neurodegenerative disorders (Dalile et al., 2019; Silva et al., 2020). Accumulating evidence suggests that SCFAs serve as crucial mediators between altered gut microbiota and cognitive impairment (Matt et al., 2018). Furthermore, SCFAs play a pivotal role in the pathophysiology of PD. SCFA deficiency has been demonstrated to increase intestinal permeability, subsequently facilitating the exposure of enteric nervous system to toxins, including lipopolysaccharides and pesticides. This cascade of events potentially leads to abnormal aggregation of alpha-synuclein protofibrils (Hirayama and Ohno, 2021). MaAsLin3 did not identify any significantly differential microbiota between the two groups under default FDR-adjusted thresholds, which aligns with the findings of Aho et al. (2024), who reported no significant gut microbiome associations with mild cognitive impairment in Parkinson’s disease using similar stringent statistical approaches. However, when the screening criteria were adjusted to raw *p*-values, *g__Senegalimassilia* exhibited significant enrichment in the PD-MCI group. Importantly, *g__Senegalimassilia* was also identified as a PD-MCI-enriched differential microorganism in LEfSe analysis, lending additional support to this finding. The null results from MaAsLin3 may be attributed to several factors. First, the predominance of low-abundance genera among differential microbiota in this study may have been masked by MaAsLin3’s stringent handling of zero-inflation issues in microbiome data. This interpretation aligns with our beta-diversity findings: while PCoA analysis across all three groups showed no significant separation using weighted UniFrac distance, unweighted UniFrac revealed significant compositional difference. This pattern suggests that microbial differences across our study cohorts primarily involve presence/absence variations or shifts in low-abundance taxa rather than changes in high-abundance community members. Such subtle compositional alterations are precisely the type that stringent statistical approaches like MaAsLin3, while enhancing rigor and reducing false positives, may be overly conservative in detecting. Second, ethnic and dietary variations between study populations may contribute to heterogeneity in gut microbiome composition and its associations with cognitive phenotypes. The discordance between our findings and those of Aho et al. may reflect population-specific microbial signatures. We will expand our sample size in future studies and conduct multi-center longitudinal investigations to further elucidate the microbial distinctions between PD-MCI and PD-NC and validate these preliminary observations.

To understand the role of plasma metabolites in PD cognitive impairment, we analyzed plasma metabolites between the two groups. Through KEGG enrichment analysis, we found 7 metabolites enriched in 3 metabolic pathways. Phenylalanine metabolism contained phenylacetylglutamine, 2-hydroxycinnamic acid, N-acetyl-L-phenylalanine, phenylacetylglycine; PPAR signaling pathway included 8-hydroxy-5Z,9E,11Z,14Z-eicosatetraenoic acid; and choline metabolism in cancer contained PC(18:1(11Z)/18:3(6Z,9Z,12Z)) and LysoPC(18:3(6Z,9Z,12Z)/0:0). Phenylalanine metabolism and PPAR signaling pathway were downregulated in PD-MCI group compared to PD-NC group, while choline metabolism in cancer was upregulated in PD-MCI group compared to PD-NC. Phenylacetylglutamine (PAGIn) is the result of the conjugation of phenylacetic acid with glutamine. In essence, it is an amino acid acetylation product of phenylacetic acid (or phenylbutyric acid following beta-oxidation) (Zimmerman et al., 1989; Shockcor et al., 1996). Our findings suggest its potential involvement in the pathogenesis of cognitive impairment in PD. PAGln is a metabolic derivative of phenylalanine (Wild et al., 2019). Accumulating evidence indicates that dysregulated phenylalanine metabolism leads to pathological cerebral phenylalanine accumulation, which precipitates cognitive deterioration through two principal mechanisms: disruption of catecholaminergic neurotransmitter synthesis, particularly affecting dopaminergic signaling, and compromise of white matter structural integrity (Thomas et al., 2023). These neurochemical and structural alterations collectively contribute to cognitive dysfunction (Romani et al., 2017; Walkowiak et al., 2023). Furthermore, PAGln modulates the GPCR signaling pathway by interacting with alpha2A-, alpha2B-, and beta2-adrenergic receptors (ADRs), thereby influencing the downstream cAMP signaling pathway (Romano et al., 2023). Studies have demonstrated that activation of beta2-adrenergic signaling mitigates the deleterious effects of Aβ accumulation on the nervous system. Given that Aβ accumulation represents a key pathological hallmark of cognitive impairment in PD (Li et al., 2013). The PD-NC group exhibited elevated levels of phenylacetylglutamine compared to the PD-MCI group, suggesting potential impairment of phenylalanine metabolism in the PD-MCI group, which could result in phenylalanine accumulation and ultimately contribute to cognitive decline. Lower PAGln levels observed in the PD-MCI group may indicate diminished resilience to Aβ neurotoxicity, potentially exacerbating cognitive dysfunction. ROC analysis revealed that phenylacetylglutamine (PAGln) exhibited robust diagnostic capability (AUC = 0.8222), suggesting its potential utility as a promising biomarker. While recent studies have established elevated plasma PAGln as a characteristic feature in AD (Yang et al., 2024b), our findings substantially extend these observations to PD, suggesting a common metabolic signature in neurodegenerative cognitive decline. This parallel between AD and PD, two disorders sharing fundamental pathological mechanisms, highlights PAGln as a potential universal mediator of cognitive deterioration in neurodegenerative conditions (Aarsland et al., 2021).

In terms of correlation between plasma metabolites and gut microbiota, regarding the 7 differential metabolites identified through KEGG enrichment analysis, we discovered multiple correlations. Among them, phenylacetylglutamine shows significant positive associations with *g__Lachnoclostridium* and *g__norank_f__norank_o__Oscillospirales*. Both genera are commonly recognized as SCFA-producing organisms. Suggesting they might participate in phenylalanine metabolism, thereby affecting plasma PAGln levels. Additionally, besides the 7 metabolites identified in the KEGG enrichment analysis pathways, other differential metabolites annotated in KEGG pathways also showed significant correlations with enriched microorganisms in each group. Among them, Multiple SCFA-producing genera (*g__Blautia, g__Lachnoclostridium, g__Sellimonas,* and *g__norank_f__Oscillospiraceae*), as well as *g__Eggerthella,* exhibited significant negative correlations with uridine 2′,3′-cyclic phosphate, which is involved in pyrimidine metabolism. Research indicates that uridine 2′,3′-cyclic phosphate is a precursor to uridine, which can improve motor symptoms in PD patients (Mironova et al., 2024). The study found that plasma levels of uridine 2′,3′-cyclic phosphate were higher in PD-MCI patients compared to PD-NC patients. While these correlation patterns suggest microbiota-metabolite interactions may play a role in PD-associated cognitive impairment, the specific underlying mechanisms require further experimental validation through mechanistic studies.

There are some limitations to our study. First, the small sample size may affect the MaAsLin3 model results. To address this, we will recruit larger cohorts from multiple centers in future research to validate our findings and improve the reliability of multivariable association analyses. Second, although our study provides important evidence for understanding the gut-brain axis mechanisms in the pathogenesis of PD-MCI, the cross-sectional design limits our ability to directly observe the dynamic effects of gut microbiota and metabolites on disease progression over time. We plan to conduct long-term follow-up of the subjects enrolled in this study with repeated sampling at multiple time points in future research, which will enable us to further elucidate the temporal role of the gut-brain axis in PD-MCI development. Third, although significant associations between microbial taxa and plasma metabolites were observed, their molecular mechanisms remain unclear. In future research, we will conduct mechanistic studies such as fecal microbiota transplantation in mice to verify causality, and *in vitro* validation of metabolites to clarify causal relationships between microbiota-metabolite pairs and cognitive decline. Furthermore, we will explore the therapeutic potential of microbiota modulation and integrate multi-omics data with clinical parameters to develop more precise diagnostic and prognostic tools.

## 5 Conclusion

Our multi-omics study identified distinct gut microbiota and metabolite profiles in PD patients with cognitive impairment. Multi-algorithm validation identified *g__Eggerthella* as the most statistically consistent microbial alteration, accompanied by altered abundance of SCFA-producing genera. Phenylacetylglutamine showed diagnostic potential (AUC = 0.8222), with altered phenylalanine metabolism correlated with microbial changes. These findings highlight altered gut-brain axis signaling in PD-related cognitive decline through microbiota-metabolite interactions, suggesting that these microbial and metabolic markers may serve as targets for further investigation in PD management.

## Data Availability

The datasets presented in this study can be found in online repositories. The names of the repository/repositories and accession number(s) can be found at: https://www.ncbi.nlm.nih.gov/, PRJNA1159471; https://www.ebi.ac.uk/metabolights/, MTBLS11094.
