# Supplementary material for: Alterations in gut microbiota and plasma metabolites: a multi-omics study of mild cognitive impairment in Parkinson’s disease
Source: Front Neurosci. 2025 Dec 1;19:1667331. doi: 10.3389/fnins.2025.1667331 (PMC12702881; doi:10.3389/fnins.2025.1667331)
Supplement: Supplementary file 1 [file Data_Sheet_1.DOCX]

Supplementary Material

# Supplementary Figures and Tables

## Supplementary Figures


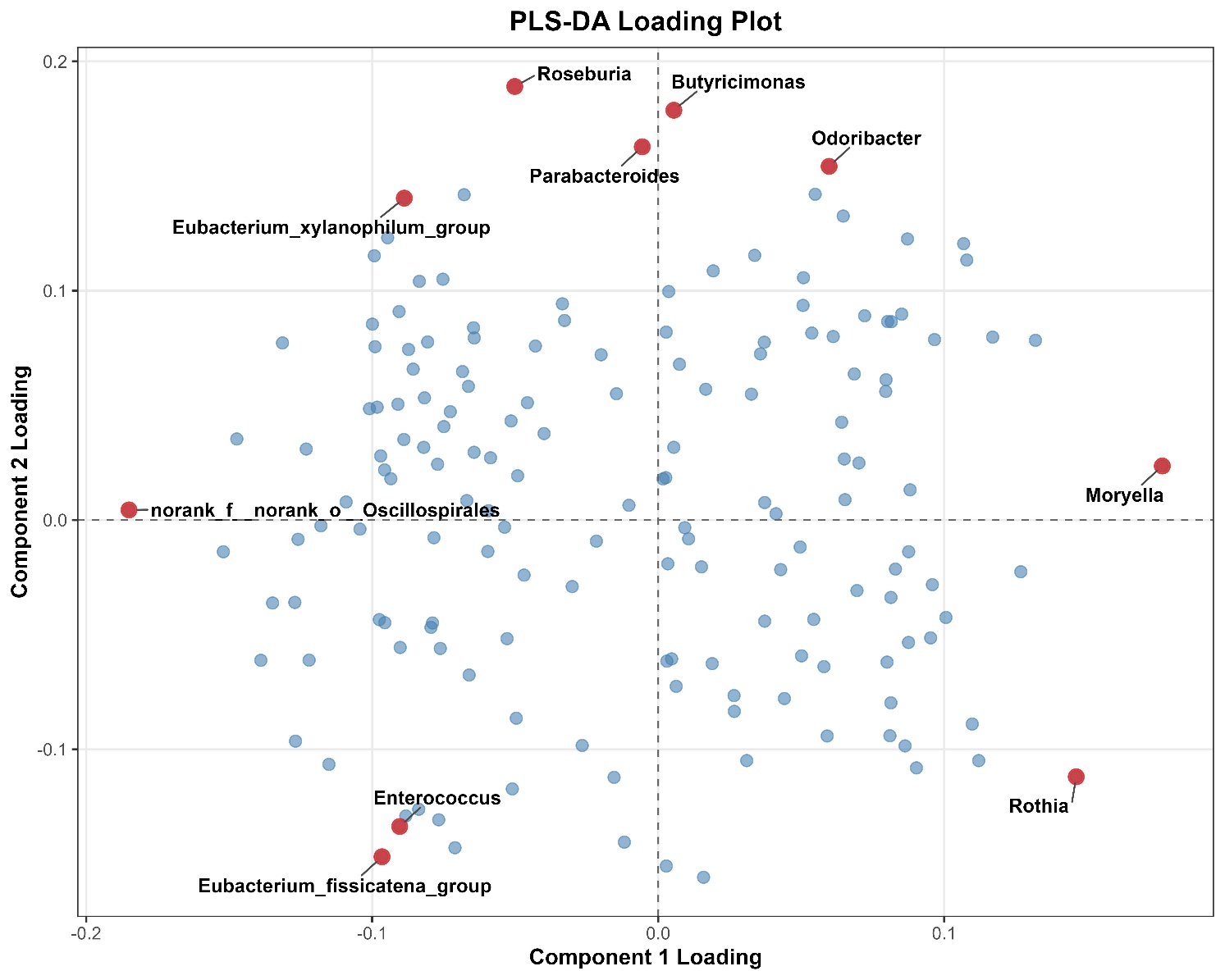


FigureS1: Loading values of gut microbial taxa (genus level) on Component 1 (x-axis) and Component 2 (y-axis). Red dots: top 10 taxa with the highest absolute loading values; Blue dots: remaining taxa. Taxa positioned at the extremes of either component represent the most discriminative features between PD-MCI and PD-NC groups.


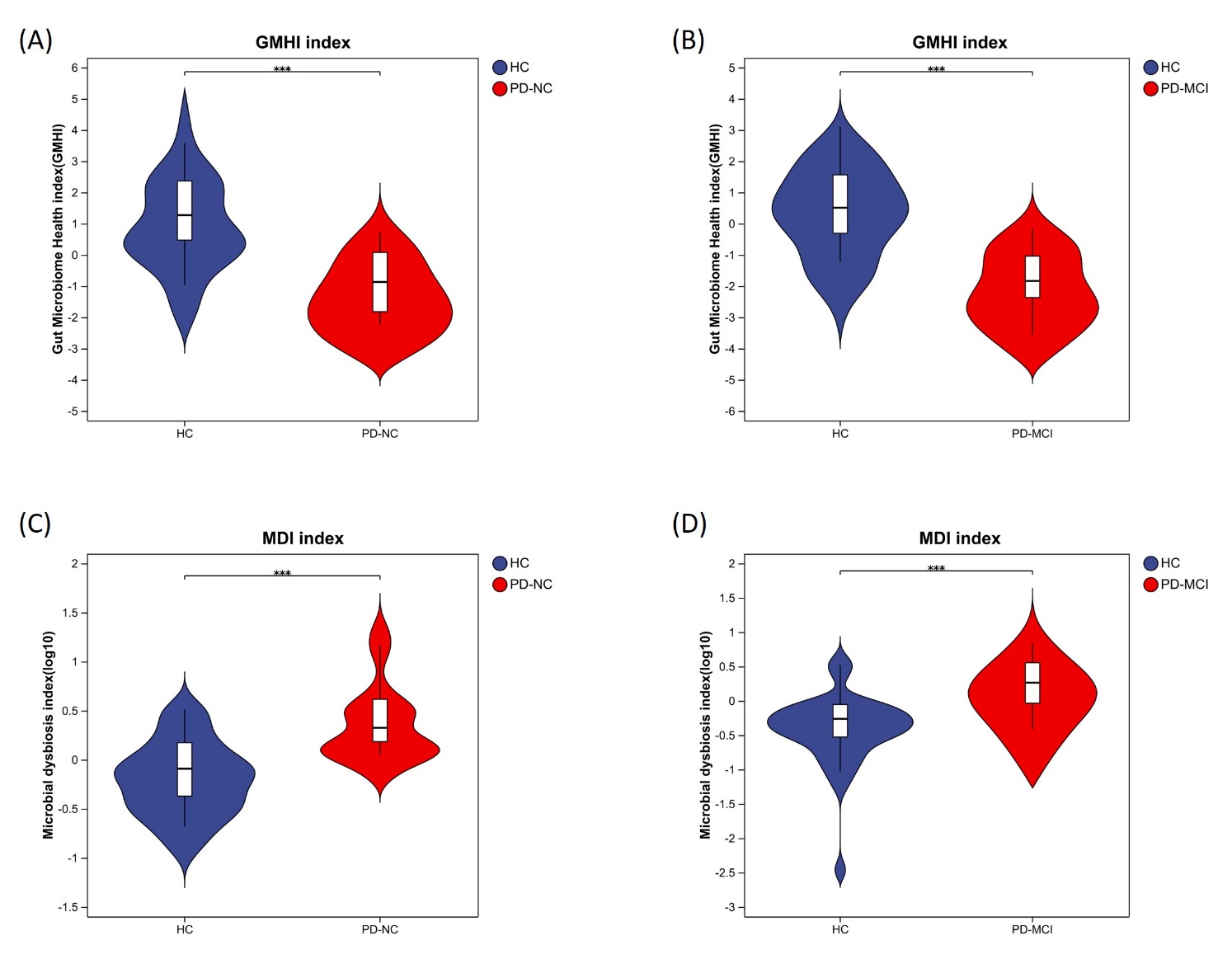


FigureS2: (A) (B) GMHI comparisons showing HC group with significantly higher scores than both (A) PD-NC and (B) PD-MCI groups (adjusted P < 0.001). (C) (D) MDI comparisons demonstrating significantly elevated values in (C) PD-NC and (D) PD-MCI groups compared to HC (adjusted P < 0.001). Violin plots with embedded box plots showing distribution, median, and interquartile range.

***adjusted P < 0.001

## Supplementary Tables

TableS1 MaAsLin3 Output

| **feature** | **coef** | **pval_individual** | **qval_individual** |
| --- | --- | --- | --- |
| *g__Senegalimassilia* | 1.978733 | 0.010632823 | 1 |
